# “Let’s get Wasted!” and Other Apps: Characteristics, Acceptability, and Use of Alcohol-Related Smartphone Applications

**DOI:** 10.2196/mhealth.2709

**Published:** 2013-06-25

**Authors:** Emma R Weaver, Danielle R Horyniak, Rebecca Jenkinson, Paul Dietze, Megan SC Lim

**Affiliations:** ^1^Burnet InstituteCentre for Population HealthMelbourneAustralia; ^2^Monash UniversityDepartment of Epidemiology and Preventive MedicineMelbourneAustralia

**Keywords:** alcohol drinking, young adult, mobile phone, applications

## Abstract

**Background:**

Smartphone applications (“apps”) offer a number of possibilities for health promotion activities. However, young people may also be exposed to apps with incorrect or poor quality information, since, like the Internet, apps are mostly unregulated. Little is known about the quality of alcohol-related apps or what influence they may have on young people’s behavior.

**Objective:**

To critically review popular alcohol-related smartphone apps and to explore young people’s opinions of these apps, their acceptability, and use for alcohol-related health promotion.

**Methods:**

First, a content analysis of 500 smartphone apps available via Apple iTunes and Android Google Play stores was conducted. Second, all available blood alcohol concentration (BAC) apps were tested against four individual case profiles of known BAC from a previous study. Third, two focus group discussions explored how young people use alcohol-related apps, particularly BAC apps.

**Results:**

384 apps were included; 50% (192) were entertainment apps, 39% (148) were BAC apps, and 11% (44) were health promotion and/or stop drinking–related apps. When testing the BAC apps, there was wide variation in results, with apps tending to overestimate BAC scores compared with recorded scores. Participants were skeptical of the accuracy of BAC apps, and there was an overall concern that these apps would be used as a form of entertainment, further encouraging young people to drink, rather than reduce their drinking and risk taking.

**Conclusions:**

The majority of popular alcohol-related apps encouraged alcohol consumption. Apps estimating blood alcohol concentration were widely available but were highly unreliable. Health departments and prominent health organizations need to endorse alcohol smartphone apps that are accurate and evidence-based to give specific apps credibility in the ever-expanding market of unregulated apps.

## Introduction

Excessive alcohol use is a major public health problem that stems partly from a social and cultural acceptance of alcohol consumption [1] despite the harms of excessive drinking being widely understood [2]. Social acceptability is exacerbated by the prevalence of alcohol marketing and promotion; young people are particularly vulnerable to regular exposure to alcohol marketing through the mass media [3]. Evidence suggests that exposure to alcohol advertisement campaigns is associated with an earlier onset of drinking and an increased level of consumption [4-6]. As well as traditional forms of advertisement, such as television and print, alcohol is increasingly being promoted through digital platforms such as social media (eg, Facebook, Twitter) [3].

Young people are prolific users of digital technologies [7]. Smartphones have revolutionized mobile communication technology by offering users Internet access and computerized functions on their mobile phones. Smartphones allow users to download applications (“apps”)—programs that are designed specifically for smartphone operating systems. In 2012, Apple reported that over 25 billion apps had been downloaded from its Apple iTunes store, and Google Play (Android’s app store) reached 15 billion downloads [8,9].

While recent evidence suggests smartphone apps offer a number of possibilities for health promotion activities [10-13], young people may also be exposed to apps with incorrect or poor quality information, since, like the Internet, apps are mostly unregulated. Little is known about the quality of alcohol-related apps or what influence they may have on young people’s behavior. A comprehensive literature search identified only one published study investigating alcohol-related apps available in the Apple iTunes store. This study analyzed available apps that addressed alcohol use, treatment, and recovery and found that while a number of apps encouraged alcohol use, few addressed behavior change; this study did not look in detail at the use or acceptability of alcohol-related apps [14]. Studies of apps in other health areas have also identified apps providing health information of varying accuracy to users [12,13,15,16]. The purpose of the current study is to review the most popular alcohol-related smartphone apps and to explore young people’s opinions of these apps.

## Methods

### Phase 1: Descriptive Analysis of Smartphone Apps

The first phase of the three-phased mixed method approach involved a content analysis of smartphone apps. The term “alcohol” was used to search Apple iTunes and Android Google Play stores in April 2012 using the stores’ default search algorithms. The top 250 apps from each store were included. The following data were extracted for each app from the stores: category as defined by the app store (eg, medical, education, entertainment, health and fitness, lifestyle), ranking (position in search results), user star rating (eg, the average number of stars given to apps by users), cost, and seller name.

A coding scheme was developed that categorized the framing, focus, purpose, consequences, attitude, and recommendations of each app. This coding scheme was developed specifically for this study, using a recent study on media reporting of illicit drugs as a template [17].

Each app was downloaded to a smartphone and reviewed by a researcher (EW) between July and November 2012; 20% of apps were reviewed by a second researcher (ML) to check for coding consistency; 9% had a coding discrepancy.

Apps were classified according to their overall purpose: those that calculated a score for the amount of alcohol in the blood or breath were classified as “Blood Alcohol Concentration (BAC) apps”, apps that provided health information or supported reducing drinking were classified as “health promotion or antidrinking apps”, and all other apps, including drinking games, cocktail recipes, and bar-finders, were classified as “entertainment apps”.

### Phase 2: Testing of BAC Apps

The second phase of the study involved testing the accuracy of BAC apps with reference to the Australian legal limit for driving (0.05% BAC). Apps were tested using data collected from a prior field-based study conducted in Melbourne. The Patron Offending and Intoxication in Night-Time Entertainment Districts (POINTED) study aimed to measure drinking practices of patrons in entertainment precincts across five Australian cities and included BAC collection [18]. Data from four randomly selected POINTED participants (including gender, age, number of drinks consumed, and hours spent drinking) were entered into each app to calculate a blood alcohol reading. Participants’ height and weight were not recorded as part of the POINTED study, so these were estimated using average values for an Australian male or female at that age [18,19]. Scores from each app were recorded and compared to the actual measured scores for each participant from the field-based study when a standardized Breathalyzer was used.

Descriptive analysis was conducted and 95% confidence intervals were determined using Stata 11.

### Phase 3: Focus Group Discussions With Young Smartphone Users

The third phase of the study was informed by the content analysis of smartphone apps and the testing of the accuracy of BAC apps. Two focus group discussions explored how young people engage with alcohol-related apps. Approval was granted by the Alfred Hospital Human Research Ethics Committee.

Participants were recruited from a cohort of festival attendees involved in a previous study about risk-taking behavior [20] who had agreed to be contacted for involvement in further research. Participants could also refer friends to participate. All participants were aged between 18 and 30 years old, drank alcohol at least occasionally, had their own smartphone, and provided written consent to participate. Participants were provided with refreshments during the focus group and reimbursed AU$40 for their time and travel costs.

Two focus groups were conducted, with a total of 12 participants (5 males and 7 females). Focus groups were held in a private meeting room at an urban Melbourne site with 2 researchers present and lasted between 60 and 90 minutes.

A focus group schedule was developed to explore participants’ opinions of alcohol-related apps and what, if any, impact they believed the apps would have on young people’s behavior. Participants were asked about previous use of apps and their opinions of these apps in general. Participants were asked to test a selection of BAC apps identified in Phase 1 of the study (by entering details such as their gender, age, and number of drinks typically consumed) and to then reflect on their usability, trustworthiness, accuracy, and how they believed they would be received by other young people. Finally, they were asked whether they thought an alcohol health promotion app would be an effective way of engaging with young people about risky drinking behavior and if so, what sort of information would be useful to them in an app.

Focus groups were audio recorded and transcribed. Interview transcripts were managed using NVivo 10 and were analyzed thematically.

## Results

### Descriptive Analysis of Smartphone Apps

**Figure 1:**
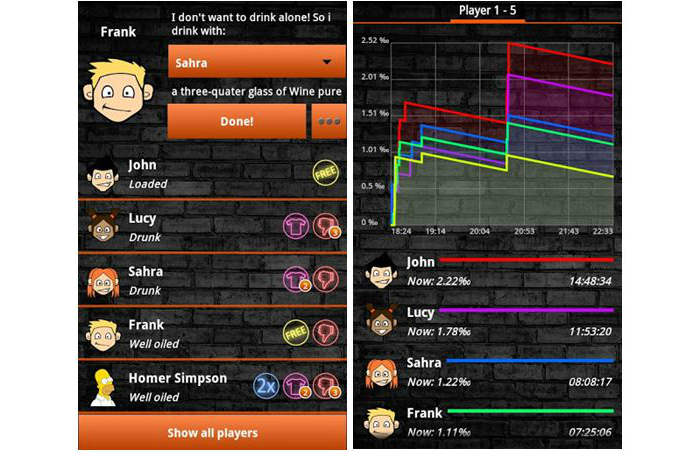
Let’s Get Wasted! screenshots showing the way that data are entered and interpreted in the entertainment app.

**Figure 2:**
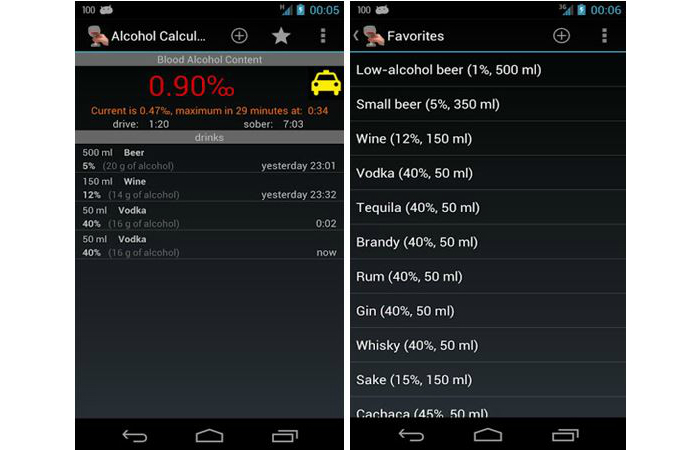
BAC screenshots showing a list from which users choose their drink and its alcohol content and how the BAC score is presented to users.

**Figure 3:**
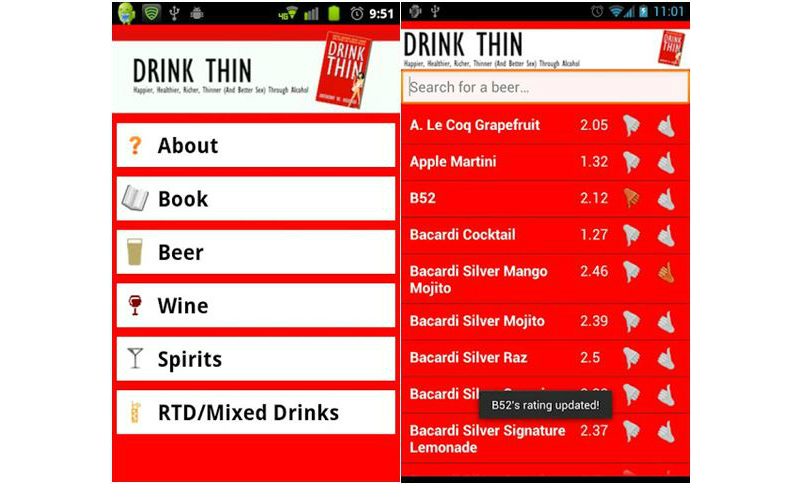
Drink Thin screenshots demonstrate the quality of information provided from an app categorized as a health promotion app.

**Figure 4:**
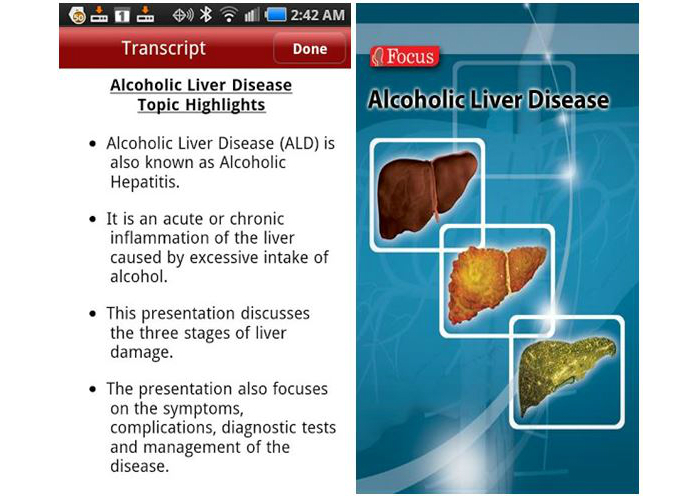
Screenshots from the Alcohol Liver Disease app demonstrating the content presented in health promotion apps.

Of the 500 alcohol-related apps, 36 were considered not relevant (ie, they did not have an alcohol focus), 52 were no longer available to download or were not compatible with the study phones, and 28 were duplicates. Of the 384 remaining apps, entertainment apps were the most common (50%, n=192), followed by BAC apps (39%, n=148), and health promotion and/or stop drinking–related apps (11%, n=44) (Table 1). Of the 192 entertainment apps, 67 (35%) were drinking games, 60 (31%) were drink-making recipes, 17 (9%) were bottle shop/bar finder apps, 13 (7%) were brewing or collectors tools, and the remaining 35 (18%) included alcohol-related jokes, brand-specific apps, other entertainment (eg, ringtones), or hangover advice. Of the 43 health promotion and/or stop drinking–related apps, 23 (53%) had a health promotion/information focus (eg, provided information on the effects of alcohol on the body, outlined alcohol laws, or described detoxing) and 20 (47%) were hypnosis or motivational apps to help with stopping drinking. The hypnosis apps used audio recordings to encourage the user to relax while simultaneously delivering persuasive antidrinking messages. Screenshots of some example apps (Let’s Get WASTED! by DDW!; Blood Alcohol Calculator by CITYJAMS; Drink Thin by 2099, LLC; and Alcohol Liver Disease by EXPANDED APPS, INC) from each category are shown in Figures 1-4.

**Table 1:** Description of alcohol-related apps.

Variable		Blood alcohol concentration (BAC)	Health promotion	Entertainment	Total
Number of apps, n (%)		148 (39)	44 (11)	192 (50)	384 (100)
**Store**					
	Android	100 (68)	32 (73)	75 (39)	207 (54)
	Apple	48 (32)	12 (27)	117 (61)	177 (46)
**Attitude towards alcohol**					
	Positive	10 (7)	1 (2)	86 (45)	97 (25)
	Negative	2 (1)	34 (78)	4 (2)	40 (10)
	Neutral	136 (92)	9 (20)	102 (53)	247 (65)
**Cost of app**					
	Free	104 (70)	19 (43)	130 (68)	253 (66)
	< $1	23 (16)	9 (21)	34 (18)	66 (17)
	≥ $1	21 (14)	16 (36)	28 (14)	65 (17)
**App store category**					
	Entertainment	31 (21)	1 (2)	39 (20)	71 (18)
	Games	0 (0)	0 (0)	20 (10)	20 (5)
	Health and Fitness	46 (31)	14 (32)	9 (5)	69 (18)
	Lifestyle	36 (24)	11 (25)	67 (35)	114 (59)
	Medical	5 (3)	9 (15)	1 (1)	15 (4)
	Tools	6 (4)	0 (0)	1 (1)	7 (2)
	Utilities	10 (7)	1 (2)	4 (2)	14 (4)
**User star rating**					
	Unrated	37 (25)	23 (53)	71 (37)	128 (33)
	1-3	57 (39)	6 (14)	58 (30)	124 (32)
	4-5	54 (36)	15 (34)	63 (33)	132 (34)

Entertainment apps were more likely to be on Apple (61%) than Android (39%); BAC apps were more likely to be on Android (68%) than Apple (32%). Most apps on Android were free to download (64%), whereas 65% of Apple apps had a cost associated with downloading. While it was not possible to determine the country of origin for most apps, the most common country of origin identified was the United States (9%).

The most common negative alcohol-related consequence mentioned was physical health (9% of all apps), followed by mental health (8%), crime (4%), and social consequences (3%). Other negative consequences described in the apps were weight gain, death, road trauma, reputation, and financial. The most common positive consequence mentioned for drinking alcohol was the social aspect (12%), followed by taste (8%). Recommendations were rarely expressed by apps, but the most frequent were to not drink (3%), to reduce drinking (2%), and to wait before driving (2%). Twenty-nine per cent (n=111) of all apps and 46% of BAC apps (n=68) had disclaimers generally stating that the app was for entertainment purposes only or that the information provided was an approximation only. See Table 2.

**Table 2:** Data extracted from each app.

Variable		Description
**All apps**		
	Country of origin	Free text (if known)
	Main focus (only those classified as “alcohol” were reviewed further)	Alcohol; Duplicate (of an already reviewed app); Unavailable (or could not be downloaded); Other (Other classified as apps that merely mentioned alcohol in their content but did not have an alcohol focus)
	Alcohol type	Beer; Wine; Spirits; General; Non-specific; Other
	Presence of any disclaimer	Free text
	Purpose of app	Alcohol brand specific; Alcohol health promotion info; Alcohol health service info; Alcohol-related jokes; Alcohol-related facts; Alcohol units consumption tracker; Blood alcohol content calculator; Brewing or collection tool; Drink-making recipes; Drinking games; Stop drinking tool; Alcohol health promotion information; Find bar/bottle shop; Other advice (eg, legal); Hangover advice
	Negative consequences	Criminal; Death; Dependence; Financial; Hangover; Physical; Mental; Violence; Sexual; Road Trauma; Other
	Positive consequences	Fun; Taste; Social; Health; Other
	Overall attitude to alcohol	Positive; Negative; Neutral
	Recommendations	Don’t drink; Reduce drinking; Don’t drive; Wait before driving
**BAC apps**		
	Variable input	Gender; Age; Weight; Hours; Number of drinks; Physical activity; Food and Water; Other
	Drink measurement	By alcohol volume; By alcohol content and volume; By unspecified “standard drink”; By specified “standard drink”; By type and volume of drink; By unspecified “Number of drinks”

### Testing of BAC Apps

A total of 98 BAC apps were used to test scenarios; 50 BAC apps were not included due to being a duplicate, unavailable, or not operational. Variables required to produce a reading varied across apps: 94% of all apps required gender, weight, and number of drinks. In addition, 68% required length of drinking session, 15% required age, 7% required information about food or water consumption, and four apps asked about recent physical exercise. Forty per cent of BAC apps measured drinks consumed by alcohol content and volume (eg, one 100 mL drink with 10% alcohol content), 31% by unspecified “number of drinks”, 19% by type and volume of drink (eg, two glasses of beer), and 14% by “standard drinks”. Fifteen apps purported to estimate BAC using a fingerprint scanner or asked users to blow air on the phone.

When entering the profile information extracted from the POINTED study, we found that different BAC apps gave a very wide range of BAC results (Table 3); this is likely due to different algorithms being used [21]. For example for Profile 1, BAC estimates ranged between 0.001 and 0.91. Most overestimated the BAC for each scenario. For example, Profile 1 involved an 18-year old male who reported having five standard drinks over a 2-hour period. In the field, this participant recorded a BAC of 0.03; when this information was entered into the BAC apps used, a mean BAC score of 0.148 (95% CI 0.118-0.178) was produced. Eighty-nine per cent of apps gave Profile 1 a higher BAC than the calibrated Breathalyzer. BAC apps that collected more data showed greater accuracy—in Profile 1, apps that required gender, weight, and number of drinks had a mean BAC score of 0.59 (SD 1.7), whereas those that required these inputs and hours of drinking had a mean BAC score of 0.30 (SD 0.32).

**Table 3:** Blood alcohol content calculator app scores using four profiles from the POINTED study.

Variable		Profile 1	Profile 2	Profile 3	Profile 4
**Profile characteristics**					
	Sex^a^	male	female	female	male
	Age^a^	18	23	19	39
	Estimated weight, kg	70	60	58	82
	Length of drinking session, hrs^a^	2	4	1	4
	Type of drinks consumed^a^	white spirits	wine + cider	punch	dark spirits
	Number of standard drinks^a^	5	16	6	7
	Recorded BAC^a^	0.03	0.05	0.08	0.11
**App testing**					
	Number of apps entered into^b^	98	96	95	93
	Mean BAC ^c^	0.148	0.648	0.220	0.163
	95% confidence intervals^c^	0.118-0.178	0.359-0.937	0.186-0.254	0.129-0.197
	Median BAC ^c^	0.102	0.405	0.19	0.122
	Minimum ^c^	0.001	0.04	0.0002	0.0002
	Maximum ^c^	0.91	13.476	1.0179	0.85
	Standard deviation	0.149	1.426	0.165	0.164
	Apps giving score higher than calibrated Breathalyzer^a^, n (%)	87 (88.8)	90 (93.8)	81 (85.3)	49 (52.7)
	Apps giving score over the legal driving limit ^d^, n (%)	83 (89.2)	90 (98.9)	84 (93.3)	76 (86.4)

^a^Information self-reported in a Melbourne field-based study. BAC calculated by a calibrated Breathalyzer.

^b^Number of BAC apps varies for each profile as some apps had restrictions on how they could be used. For example, if you first entered your profile as a male, you were unable to change the profile to be female; multiple users could not use the app at the same time due to a time delay.

^c^Data calculated by BAC apps.

^d^Limit for legal driving in Australia is 0.05.

### Focus Group Discussions With Young Smartphone Users

#### Health and Alcohol-Related App Use Among Focus Group Participants

Focus group participants generally had experience using health-related apps, with exercise apps such as tracking your run/cycle and gym workout being the most popular. Many participants had also used alcohol-related apps, all of which fell into the entertainment category (including bar trackers, bottle shop finders, drinking games, “happy hour” finders, and cocktail recipe apps). No participants reported personal use of BAC apps, although when prompted, 2 participants reported that they had seen them used by friends.

#### Utility of BAC Apps

Young people generally viewed BAC apps as games or forms of entertainment rather than as health promotion or education tools. One suggested that these apps would encourage competition between friends and possibly encourage them to get drunk: “If I had one of them, I’d be with my mates and it would be like ‘who can get the highest score?’ You’d just get smashed!” Participants were, however, receptive to the idea of alcohol-related apps and felt that they could serve a variety of useful purposes, such as preplanning for a night out or informing decisions about whether it was safe to drive. One participant commented: “If you’re just using it to see if you can drive or not, which means that you won’t be that drunk, and you kind of have the capacity to put it all in accurately enough to get a good idea of whether or not you should be driving—that’s good.”

Despite this, issues of trust and skepticism were raised by participants. Participants recognized that the apps were not necessarily accurate, noting that “there are so many confounders...like it’s not just weight and height.” The difficulties of accurately monitoring alcohol consumption were also noted: “It’s hard for it to be completely accurate, unless you’re tracking ‘I had this and it has this much alcohol in it, and I’ve had this and it has this much alcohol in it’, because no one measures standard drinks when you’re out.” Furthermore, some participants were wary of the potential negative effects, where people would intentionally drive because of app results, even when they knew they should not. One participant described an experience where a friend had used a BAC app and then driven home: “It was so bad.” Another participant stated: “People would just use it as a way to justify their actions.”

The overall concern among participants was that BAC apps would encourage young people to drink, rather than reduce their drinking. One participant stated: “I think it’s a bit dangerous, as afterwards it gives you your blood alcohol content, you can drink more and it tells you how much more you can drink, until you can drive so that’s wrong. If you follow that, you’re in trouble.” Some were concerned that the information provided would mislead young people, as highlighted when one participant stated: “If you’re using it and you have a low blood alcohol, and you’re already pretty drunk, you’d be like, ‘I’ve got low blood alcohol. I can just keep drinking’ or ‘I can drive home’…If it’s inaccurate, it can just be really dangerous.”

When testing BAC apps, participants generally thought apps that were preprogrammed with type of drink (eg, beer/wine) were easier to use than those asking for a specific volume or percentage of alcohol. Some found the apps difficult to use because of imperial rather than metric measurement, the detail of information required, or the layout of the app. One participant commented: “We couldn’t figure out how to end the drinking session [in the app]. And we’re sober and trying to figure out how to use the app and so what are you going to do when you’re already drinking?!”

#### Suggestions for Future Health Promotion Apps

Safety while drinking alcohol was the key concern among focus group participants, and apps were identified as being able to play an important role in promoting this. One participant stated, “It’s just about keeping safe while you’re drunk” and another suggested that an app that was like “a little book of how to get out of drunken situations” could be helpful. Participants identified that an app offering essential services to young people “when you’re panicked” is what is needed. Apps that could provide information on “where good spots are to get cabs” or in situations where “friends have had too much to drink…and you don’t know what to do” would be useful. Sexual health information, referrals to appropriate health services, and hangover advice were also areas of interest for a new health promotion app. One participant thought an app that had both a benefit to people while drinking as well as a longer-term benefit, such as an app that told you to “keep drinking water—don’t be hung over in the morning”, would be of interest. Participants felt there was a need for such an app as this kind of information is not provided at school, and an Internet connection is often not reliable when in a nightclub so having an app that does not rely on the Internet ensures people have the information with them.

## Discussion

### Principal Findings

To date, only one other study has been published that explored alcohol-related apps [14]; the current study is the first study to our knowledge to critically review alcohol-related apps on both Android’s Google Play and Apple’s iTunes stores. This is also the first study to specifically study the accuracy of BAC apps and to explore their use and acceptability among young people. Our study found that half of alcohol-related apps reviewed were classified as entertainment apps that endorsed drinking, 30% were BAC apps, and a minority (11%) were classified as health promotion apps.

Testing BAC apps suggested that these apps not only overestimated BAC level but also provided an extremely wide variation in scores. This could be because apps did not collect all the necessary data to accurately calculate a BAC level, such as height, age, and hours spent drinking, or because their method of calculation was flawed. Alcohol consumption was also not collected in a standardized manner. BAC apps that collected more data displayed greater accuracy and consistency in their scores. Some apps did not even collect data but simply provided a random BAC when users blew air onto the smartphone. Reassuringly, participants in the focus group discussions were skeptical of these apps, recognizing the number of variables that could affect their accuracy, including their own self-reporting of number of “standard drinks” consumed. Current literature suggests that the accuracy of self-reporting alcohol consumption depends on the social context in which it occurs [22,23]. Participants agreed that peer pressure would often prevent accurate or sensible use of these apps and could further fuel heavy drinking among some groups.

Worryingly, many BAC apps gave a specific time at which users were able to resume driving after a drinking session. While participants thought this was useful for planning their night out, they also identified that BAC apps failed to recognize variations between countries; it was often unclear in which country apps were developed and therefore the legal limit being referenced. Many users may have their provisional license and according to Australian law must have a BAC of 0.0% when driving [24]; hence, following the advice of these apps would potentially lead to breaking the law. Another factor influencing the accuracy of the information produced was how data were entered; participants found some apps difficult to use, such as when entering the alcohol volume consumed. Many BAC apps (54%) also had no disclaimer warning that they may not be valid or reliable. This may increase the risk of users perceiving the output to be accurate.

Entertainment apps including drink recipes, drinking games, and bar finders dominated the app stores and were the only type of app previously used by participants. Given this, it is reasonable to assume young people are more likely to download apps that encourage drinking rather than apps designed to reduce drinking and/or harm. This has also been noted as an issue in tobacco use; in a recent review of tobacco apps, researchers found that 107 pro-smoking apps were very popular, having been downloaded by over 6 million users [15]. Research has shown that drinking games encourage excessive alcohol consumption and are associated with several negative alcohol-related consequences [25]. Only 44 health promotion apps were reviewed as part of this study. Of concern is that some of these apps gave the impression of promoting health messages when in fact they were inaccurate in content. Some were categorized as “Medical” or “Health & Fitness” apps in the app stores, reinforcing their false legitimacy to users. For example, the app named “Drink Thin” (Figure 3) was categorized as a “Health & Fitness” app, despite promoting health and weight loss through drinking *more* alcohol. Poor categorization of apps, particularly of those wrongly claiming to provide legitimate health messages, was also noted by researchers investigating the pro-smoking apps [15].

### Limitations

This study had limitations. First, participants in the focus groups were a small sample with relatively homogenous characteristics and so may not represent the views of all young people. Second, the accuracy of data from the POINTED study may be subject to social desirability or recall bias, as all data, excluding the BAC score, were self-reported [18]. Finally, using the term “alcohol” to search app stores for alcohol-related apps may have limited our search but was justified given this was a pilot project and the large number of apps retrieved using one search term; multiple terms such as “drinking” could be explored in future research.

### Conclusion

In 2012, the number of people downloading health-related apps reached 247 million [26]. Smartphone apps are clearly growing in popularity and will play a key role in the future of health promotion initiatives. Studies have proven the effectiveness of using mobile phones in health promotion [10,20], and smartphone apps are shown to be effective in managing people suffering from long-term illnesses and alcohol dependence by offering support, resources, and information [27]. Health departments and prominent health organizations need to move with the current climate and endorse quality, evidence-based apps to give specific apps credibility in an ever-expanding, unregulated market, as is being done by the Australian Drug Foundation [28]. Apps developed by health professionals need to be innovative, useful, desirable, and fun in order to compete with apps encouraging unhealthy behaviors. While young people in our study displayed skepticism about the quality and accuracy of apps, emphasis should be placed on raising awareness of fraudulent or inaccurate apps. App stores could also play an important role in regulating the quality of available apps, by ensuring all apps have an appropriate disclaimer and/or age limit and are categorized appropriately.

## Abbreviations

BAC: blood alcohol concentration
POINTED: Patron Offending and Intoxication in Night-Time Entertainment Districts
